# Preoperative scallop-by-scallop assessment of mitral prolapse using 2D-transthoracic echocardiography

**DOI:** 10.1186/1476-7120-8-1

**Published:** 2010-01-01

**Authors:** Giovanni Minardi, Paolo Giuseppe Pino, Carla Clotilde Manzara, Giovanni Pulignano, Giulio Giuseppe Stefanini, Giuseppe Nicola Viceconte, Stefania Leonetti, Andrea Madeo, Carlo Gaudio, Francesco Musumeci

**Affiliations:** 1 Department of Cardiovascular Science, "S Camillo-Forlanini" Hospital, Rome, Italy; 2 Heart and Great Vessels Department "Attilio Reale", Sapienza University of Rome, Italy

## Abstract

**Background:**

This study was conducted to assess the accuracy of harmonic imaging 2D-transthoracic echocardiography (2D-TTE) segmental analysis compared to surgical findings, in degenerative mitral regurgitation (MR).

**Methods:**

Seventy-seven consecutive patients with severe degenerative MR were prospectively enrolled. Preoperative 2D-TTE with precise localization of prolapsing or flailing scallops/segments was performed. All patients underwent mitral valve surgical repair. Surgical reports (SR), including valve description, were used as references for comparisons. A postoperative control 2D-TTE was performed.

**Results:**

Out of 462 scallops/segments studied, surgical inspection identified 102 prolapses or flails (22%), 92 of which had previously been detected by 2D-TTE (90.2% sensitivity, 100% specificity). Agreement between preoperative 2D-TTE segmental analysis and SR was 97.8% (k = 0.93; p < 0.0001). Sixty-nine out of 77 2D-TTE reports were completely concordant with SR (89.6% diagnostic accuracy). None of the 8 non-concordant 2D-TTE reports were in complete disagreement with SR. P2 scallop was always involved in posterior leaflet prolapse or flail and was described correctly by 2D-TTE in 68 out of 69 patients (98,7% agreement, k = 0,93; 98.5% sensitivity). The anterior leaflet was involved in 14 patients (18%); A2 segment was involved in all of those cases and was correctly detected by 2D-TTE in 13 (98,7% agreement, k = 0,95; 92,8% sensitivity). Antero-lateral and postero-medial para-commissural prolapse or flail had a lower prevalence (14% and 10% respectively), with 2D-TTE sensitivity respectively of 64% and 50%.

**Conclusions:**

2D-TTE, performed by an experienced echo-lab, has very good diagnostic accuracy in localizing the scallops/segments involved in degenerative MR, particularly for the middle ones (P2-A2), which represent almost the totality of prolapses. More invasive, time consuming and expensive exams should be reserved to selected cases.

## Background

Echocardiographic mitral valve (MV) prolapse is defined as single or bileaflet systolic prolapse at least 2 mm beyond the parasternal long-axis annular plane, with or without leaflet thickening [1]. The prevalence is estimated at 2-3%, and it is equally distributed between men and women [2].

MV prolapse assessment follows Carpentier's widely recognised nomenclature [3,4].

The most important complication of mitral valve prolapse is severe mitral regurgitation (MR), which may result from either progressive myxomatous degeneration or chordal rupture with leaflet flail [5]. MV repair is the preferred method of treatment over MV replacement, if surgically feasible. This strategy preserves left ventricular function and decreases risk of hemolysis, thromboembolism, and hemorrhage (due to anticoagulation therapy). Suitability for MV repair can be predicted preoperatively by echocardiography, assessing mitral annular calcification and extension of valvular degeneration [6,7]. Therefore to plan surgical repair a segmental analysis of the prolapsing valve is essential [8].

Both transthoracic echocardiography (2D-TTE) and transesophageal echocardiography (2D-TEE) are valid methods in the identification of MV prolapse or flail [1]. Several studies have demonstrated that functional assessment of MR by 2D-TEE and 3D imaging is a strong determinant of valve reparability and postoperative outcome with significant incremental value over 2D-TTE [7,9-12]. However, the advent of new beam formers and harmonic imaging has immensely improved the quality of 2D-TTE; thus, the diagnostic accuracy of 2D-TTE in the evaluation of the MV needs to be re-examined. Recently Monin et al. [13] showed that functional assessment of MR by 2D-TTE can accurately predict valve reparability in patients undergoing surgery for severe MR, pointing out that in most cases preoperative 2D-TEE is not mandatory.

We have evaluated the accuracy of 2D-TTE in the assessment of prolapsing or flailing scallops/segments in a series of consecutive patients that underwent surgical repair for MR. The aim of the study was to assess, through a scallop-by-scallop analysis, the agreement between 2D-TTE and surgical report descriptions of MV scallops/segments, and the sensitivity and specificity of 2D-TTE in identifying prolapsing or flailing scallops/segments. We also evaluated the total concordance of 2D-TTE with surgical report for each patient; reports were classified as "concordant" or "non-concordant", where "non-concordant" meant incomplete agreement or disagreement.

## Methods

### Study population

Seventy-seven consecutive patients affected by isolated moderate to severe degenerative MR were prospectively enrolled at our Hospital, between September 2006 and December 2007. All patients underwent preoperative and postoperative 2D-TTE and surgical repair with direct inspection of the valve by the surgeon (reference for comparisons). Intraoperative 2D-TEE examination was performed in all patients. Exclusion criteria were severe mitral annular calcification, coexistence of other cardiac disease, including non-degenerative MR, other valvulopathy, coronary artery disease, and congenital heart disease, and suboptimal acustic window. No patient was excluded on the basis of age, sex, race, LV dysfunction, or associated non-cardiac comorbidities. This study was examined and approved by our local Advisory Board and informed consent was obtained from all patients.

### Echocardiographic analysis

**Figure 1:**
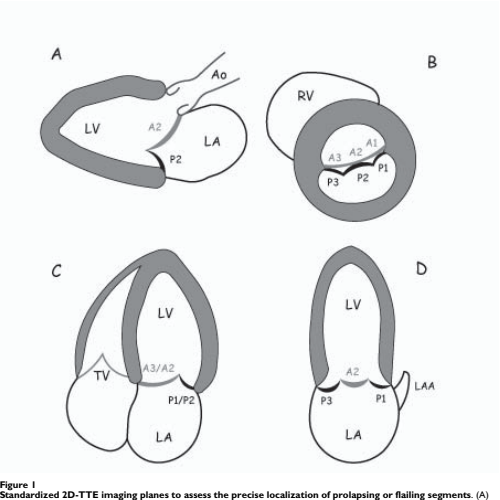
**Standardized 2D-TTE imaging planes to assess the precise localization of prolapsing or flailing segments**. (A) Parasternal long-axis view. (B) Parasternal short-axis view. (C) Apical four-chamber view. (D) Apical two-chamber view (intercommissural plane). Ao = ascending aorta; LA = left atrium; LV = left ventricle; LAA = left atrial appendage; RV = right ventricle; TV = tricuspid valve.

2D echocardiographic equipment (Sonos 5500, Philips, Eindhoven, The Netherlands) and standard guidelines for functional analysis of MR were used. A comprehensive 2D-TTE examination was performed on all patients by 4 senior echocardiographers, working in the same echo-lab, who had extensive experience in MR assessment optimised by frequent confrontation with surgical findings. MR was quantified by validated different 2D and Doppler methods [14,15]: proximal flow convergence method (PISA), mitral-aortic flow velocity integral ratio (MAVIR), vena contracta (VC), and jet area/atrium area. The grade of regurgitation was assessed on a standardized scale from 0 (none) to 4 (severe). Prolapsing or flailing leaflets were assessed according to standard criteria and described following Carpentier's functional classification with a precise localization of the involved scallops or segments, according to four standardized imaging planes (Figure 1) [13,16,17].

### Surgical analysis

Surgical findings, directly transcribed from the surgical reports, were the references to evaluate the diagnostic accuracy of 2D-TTE. The surgeons were aware of functional analysis by 2D-TTE but were not informed of the study. Thus, echocardiographic findings were independent from surgical assessment.

### Postoperative 2D-TTE control

A control 2D-TTE was performed on each patient 4 to 6 days after surgery. Systolic function, left ventricle volumes, mitral valve analysis (persistent regurgitation grade and transvalvular mean gradient) and systolic pulmonary artery pressure (PAPs; estimated through transtricuspidal gradient) were accurately evaluated. Left ventricle outflow tract (LVOT) was studied in all patients to exclude a possible dynamic obstruction.

### Statistical analysis

Continuous variables were described as means and standard deviations (SD) and categorical variables as counts and percentages. Sensitivity, specificity, and accuracy of 2D-TTE were calculated according to standard formulas. Diagnostic accuracy of 2D-TTE was defined as the sum of true positive and true negative results divided by the number of patients. The agreement between surgical findings and 2D-TTE for the localization of prolapsing scallops/segments was assessed by calculating the kappa coefficient (a value > 0.80 indicating excellent agreement), and kappa statistics were compared using the Z test. Preoperative and postoperative echocardiographic continuous variables were compared using the t-Student test. Two-tailed p values > 0.05 were considered statistically significant. Analyses were conducted using SPSS Statistics^® ^15.0 for Mac OS software.

## Results

### Baseline characteristics

Among the 77 patients, 51 (66%) were men, and the median age was 60.3 years (SD 14.3). All patient were Caucasian. Mean MR was moderately severe (grade 3.5). All patients had a normal left ventricle systolic function (mean ejection fraction 64%). Forty-two patients (54%) had a left ventricle end-diastolic diameter (LVEDD) larger than normal for age and sex. Sixty-five patients (84%) had an enlarged left atrium. The mean PAPs was 42 mmHg (SD 10.6). Baseline general characteristics and echocardiographic data are presented in Table 1.

**Table 1:** Patients baseline characteristics

Variable	Overall	Male	Female
Patients (n)	77	51 (67%)	26 (33%)

Age ± SD (yrs)	60 ± 14	59 ± 13.8	62.8 ± 15.1

Mean MV Regurgitation	3.5	3.5	3.5

Mean PAPs ± SD (mmHg)	42 ± 10.6	41 ± 9.8	49 ± 12

Mean Ejection Fraction ± SD (%)	64 ± 7.8	63.3 ± 7	64.9 ± 8.9

Mean LV End-Diastolic diameter ± SD (mm)	59 ± 7.5	61.1 ± 6.8	55,2 ± 8

Mean Left Atrium area ± SD (cmq)	28 ± 7	27,8 ± 6.3	28,5 ± 9.3

MV, mitral valve; PAPs, systolic pulmonary artery pressure; LV, left ventricle.

### Surgical Techniques

In presence of ruptured chordae of the posterior mitral leaflet, quadrangular resection of the flail segment was performed using the "sliding technique". Posterior annuloplasty completed the repair. The anterior leaflet was approached surgically only in presence of a flail area by insertion of gore-tex chordae.

### 2D-TTE vs Surgery agreement and diagnostic accuracy

A total of 462 scallops/segments were studied according to Carpentier's classification; the agreement between preoperative 2D-TTE report and surgical findings concerning the localization of prolapsing or flailing scallops/segments was 97.8% (kappa coefficient 0.93; p < 0.0001).

**Figure 2:**
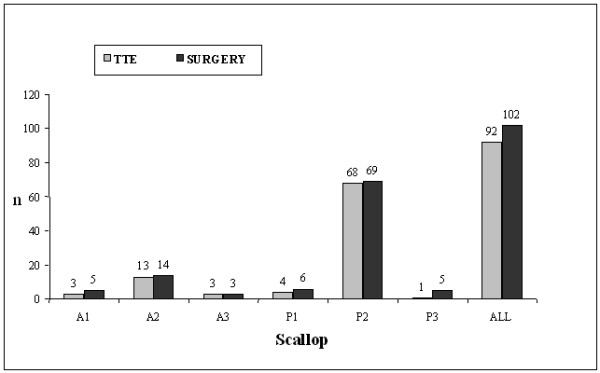
**2D-TTE vs Surgery prolapse/flail detection**.

Surgical inspection identified 102 prolapsing or flailing scallops/segments (prevalence 22%), 92 of these had previously been detected by 2D-TTE, with a sensitivity and specificity of 90.2% and 100% respectively (Figure 2).

Sixty-nine out of 77 2D-TTE reports were "concordant", meaning that the 2D-TTE report was totally concordant with the surgical description, with a diagnostic accuracy of 89.6%. The 8 2D-TTE reports classified as "non-concordant" were all partially concordant. No 2D-TTE report was in complete disagreement with the surgical findings.

### Segmental analysis

Posterior leaflet was prolapsing in 69 patients (90%), in 6 of them it was associated with anterior leaflet prolapse determining bileaflet prolapse. P2 scallop was always involved in posterior leaflet prolapse or flail and was described correctly by 2D-TTE in 68 out of 69 patients, with 98,7% agreement (kappa 0,93), 98.5% sensitivity and 100% specificity. In 80% of cases (n = 55) P2 prolapse was isolated.

The anterior leaflet was prolapsing or flailing in 14 patients (18%), A2 segment was involved in all cases and was correctly detected by 2D-TTE in 13 cases with 98,7% agreement (kappa, 0,95), 92,8% sensitivity and 100% specificity.

**Figure 3:**
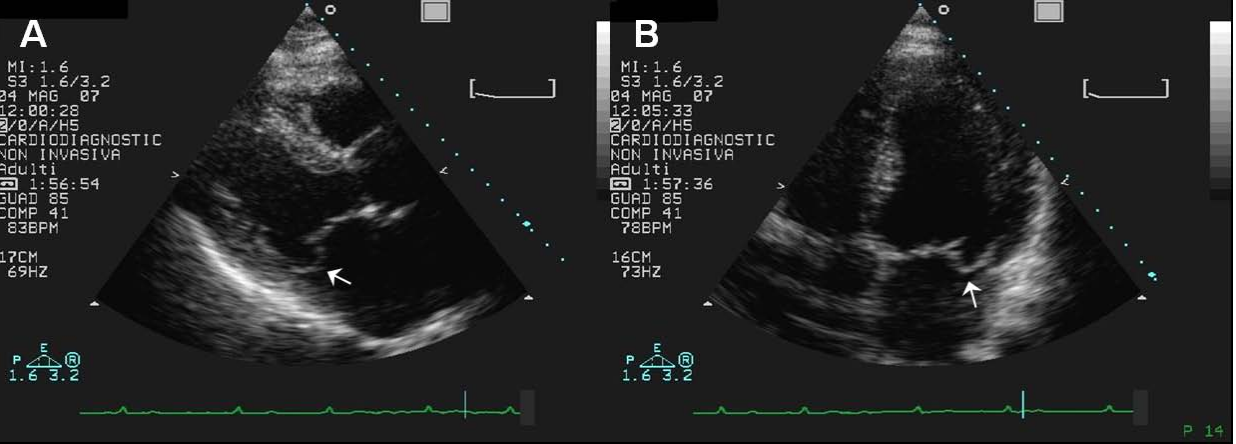
**2D-TTE parasternal long-axis (A) and apical four-chamber (B) views: mitral valve prolapse involving P2 and P1 scallops (arrows)**.

Para-commissural prolapse (P1, P3, A1, A3), always associated with middle scallop/segment (A2 and/or P2) involvement, was present in 19 patients (25%) (Figure 3). The antero-lateral (AL, A1-P1) and postero-medial (PM, A3-P3) para-commissural scallops/segments were involved in 11 and 8 cases respectively. In detecting para-commissural prolapse or flail 2D-TTE had 64% sensitivity and 100% specificity on AL scallops/segments, and 50% sensitivity and 100% specificity on PM scallops/segments.

Bileaflet prolapse was detected by 2D-TTE in 3 out of 6 cases, with 50% sensitivity and 100% specificity (Table 2).

**Table 2:** Segmental analysis

Scallops/Segments	n°	Prolapse/Flail	Prevalence (%)	TTE detection	Sensitivity (%)	Specificity (%)
All	462	102	22.1%	92	90.2	100

A1	77	5	6.5%	3	60	100

A2	77	14	18.2%	13	92.8	100

A3	77	3	3.9%	3	100	100

P1	77	6	7.8%	4	66.7	100

P2	77	69	89.6%	68	98.5	100

P3	77	5	6.5%	1	20	100

A	77	14	18.2%	13	92.8	100

P	77	69	89.6%	68	98.5	100

A+P	144	6	7.8%	3	50	100

A: anterior leaflet (A1 and/or A2 and/or A3); P: posterior leaflet (P1 and/or P2 and/or P3); A+P: bileaflet involvement.

### Clinical outcome

Mitral valve repair was successful in all patients. The postoperative 2D-TTE demonstrated a significant reduction or complete absence of MR in all patients. Mean postoperative PAPs was 34 mmHg (SD 6.6), with a 19% relative reduction (p < 0.0001). In no patient was detected a significant mitralic transvalvular mean diastolic gradient or a dynamic LVOT obstruction (Table 3).

**Table 3:** Preoperative and postoperative hemodynamic parameters

Variable	Before surgery	After surgery	Relative reduction	P value
Mean PAPs ± SD (mmHg)	42 ± 10.6	34 ± 6.6	19%	<0.00001

Mean LVEDV ± SD (ml)	144.8 ± 42.5	107.1 ± 37.2	26%	<0.00001

Mean Ejection Fraction ± SD (%)	63.9 ± 7.7	54.6 ± 8.6	14.5%	<0.00001

MV mean gradient ± SD (mmHg)	-	3.9 ± 1.5	-	-

MV, mitral valve; PAPs, systolic pulmonary artery pressure; LVEDV, left ventricle end diastolic volume.

## Discussion

Degenerative MR is the second frequently encountered valve disease in western countries [18]. Nowadays, surgical valve repair is considered the optimal therapeutic strategy for severe MR and is an incentive for early surgery in asymptomatic patients. Precise localization of involved scallops and segments in degenerative MR is a major issue of concern, given its strong influence on the rate of successful repair [19]. Therefore, an accurate preoperative assessment of mitral valve morphology and functionality is required for correct surgical planning [6,20,21]. Several studies have demonstrated the validity of 2D-TEE and 3D imaging (both 3D-TTE and 3D-TEE) in the assessment of MR, but few data are available on the diagnostic accuracy of 2D-TTE in experienced hands, since the advent of harmonic imaging [7,11,12,22]. Our study shows that 2D-TTE performed in a high flow surgical-experienced echo-lab, has strong diagnostic reliability in the assessment of MV prolaspse or flail.

Previous studies have showed the superiority of 2D-TEE over 2D-TTE in assessing functional anatomy of MR. In most previous studies, the available technology for 2D-TTE was fundamental imaging, yielding suboptimal image quality in a substantial proportion of patients [9,23]. Advance in 2D-TTE image quality due to new transducers and harmonic imaging technology [24], and the use of four standardized imaging planes for precise localization of prolapsed or flailed scallops and segments have improved the accuracy of 2D-TTE [13,17]. Monin et al. have showed an impressive reduction in non-conclusive 2D-TTE due to poor image quality (1%) [13]. In our study, no patient had non-conclusive preoperative transthoracic exam due to suboptimal image quality.

We demonstrated an overall 97.8% agreement of 2D-TTE with surgical findings in depicting scallop/segment prolapse or flail, with a kappa coefficient of 0.93, meaning an almost perfect agreement. This result is superior to the 2D-TEE vs surgical findings agreement reported by Monin et al. (93%; kappa 0.85).

Müller et al. comparing 3D-TEE to 2D-TEE assessed a high diagnostic accuracy of both techniques, with a slight superiority of the former in the identification of para-commisural and bileafleat prolapse, thus suggesting the utilization of 3D-TEE in selected cases. Sensitivity and diagnostic accuracy of 2D-TTE showed in our study are equivalent to those described by Müller et al. for 2D-TEE [7].

Our segmental analysis confirmed that the most prevalent lesion in degenerative MR is a single prolapse or flail of P2 (prevalence 89.6%), as already reported in Literature [7,12,13,19]. 2D-TTE was able to identify P2 prolapse or flail in 68 cases out of 69, with high sensitivity (98.5%) and specificity (100%). The prevalence of A2 prolapse or flail in our study was 18.2%, confirming this segment as the second most frequent lesion site. Concerning A2, 2D-TTE detected 13 out of 14 cases, with 93% sensitivity and 100% specificity. The middle portions of the leaflets (P2 and/or A2) were involved in all patients, and 2D-TTE identified 75 out of 77 cases, with 97.4 diagnostic accuracy, 97.6% sensitivity and 100% specificity. This data shows the extremely high power of 2D-TTE in assessing the middle scallop/segment involvement, which represents virtually all mitral valve prolapses or flails (Additional files 1, 2 and 3).

Overall diagnostic accuracy of 2D-TTE in evaluating every single patient was 90.9%, meaning a complete concordance in 69 out of 77 patients. In the 8 patients "non-concordant" the incomplete concordance was imputable to para-commissural scallops/segments in 6 cases.

In our study the involvement of para-commissural scallops and segments had a lower prevalence compared to middle ones. The AL and PM para-commissural areas were involved in 11 (14%) and 8 (10%) cases respectively. Bileaflet prolapse was present in 6 patients (8%). Considering the low prevalence, data regarding bileaflet and para-commisural involvement should be considered observational. Anyway, 2D-TTE sensitivity was lower in detecting prolapse or flail in these areas (AL 64%; PM 50%; bileaflet 50%) determining a relevant reduction in global diagnostic accuracy.

Recently Beraud et al. compared 3D-TTE multiplanar reconstruction to 2D-TTE, assessing that the added value of 3D-TTE was specifically significant in complex cases with para-commissural involvement [25]. Anyway also in their results the prevalence of commissural involvement was low and was associated to middle portions prolapse in 20 out of 27 cases.

We want to remark that in our study para-commissural prolapse or flail was always associated with middle portion prolapse or flail suggesting a middle-prolapse extension rather than a direct involvement of para-commissural areas. Therefore, a non-detected para-commissural involvement always determined an underestimation of complete diagnosis rather than a missed diagnosis.

## Study Limitations

1) 2D-TTE exams of our study were performed by senior echocardiographers, working in the same echo-lab, with extensive experience in MR assessment optimised by frequent confrontation with surgical findings. Thus, our result may not be generalized to less experienced centers. 2) 2D-TEE and/or 3D imaging control were not considered in the design of the study. 3) Sample size was inadequate to evaluate the diagnostic accuracy of 2D-TTE on para-commissural and bileafleat prolapse or flail.

## Conclusions

Our study shows that 2D-TTE with harmonic imaging technology, performed by senior echocardiographers, working in the same high-flow surgical experienced echo-lab, has very good diagnostic accuracy and high reproducibility in localizing the scallops and/or segments involved in degenerative MR. This is evident especially for middle area (P2 and/or A2), almost always interested in this disease.

In most cases accurate 2D-TTE analysis allows more invasive, time consuming and expensive exams to be avoided, leading to a better cost-effectiveness. This is a relevant advantage considering today's great attention to public health budget. Preoperative 2D-TEE should be limited to cases of suspected para-commissural isolated involvement and/or when more details are needed for surgical planning. 2D-TEE remains mandatory for intraoperative evaluation and monitoring.

Further studies comparing 2D-TTE to 2D-TEE and/or 3D imaging on a larger group of patients are needed to evaluate possible additional information useful for surgical planning and for a better assessment of para-commissural and bileaflet involvement.

## Competing interests

The authors declare that they have no competing interests.

## Authors' contributions

GM played a main role in the conception and design of the study, coordinated it, performed part of the 2D-TTE exams and critically revised the manuscript. GPP, CCM and GP participated in the design of the study and carried out the 2D-TTE exams. GGS and GNV participated in the design of the study, performed the statistical analysis, analyzed and interpreted data and draft the manuscript. AM, SL and CG helped to draft the manuscript. FM was responsible for surgical data and critically revised the manuscript. All authors have read and approved the final manuscript.

## Supplementary Material

Additional file 1 **2D-TTE parasternal long-axis view: mitral valve prolapse involving A2 segment and P2 scallop**. Video clip showing 2D-TTE parasternal long-axis view: mitral valve prolapse involving A2 segment and P2 scallop. Click here for file

Additional file 2 **2D-TTE parasternal short-axis view: mitral valve prolapse involving A2 segment and P2 scallop with severe mitral regurgitation (color-Doppler images on the right)**. Video clip showing 2D-TTE parasternal short-axis view: mitral valve prolapse involving A2 segment and P2 scallop with severe mitral regurgitation (color-Doppler images on the right). Click here for file

Additional file 3 **2D-TTE apical four-chamber view: mitral valve prolapse involving A2 segment and P2 scallop with severe mitral regurgitation (color-Doppler images on the right)**. Video clip showing 2D-TTE apical four-chamber view: mitral valve prolapse involving A2 segment and P2 scallop with severe mitral regurgitation (color-Doppler images on the right). Click here for file
